# Author Correction: Green synthesis of graphene oxide by seconds timescale water electrolytic oxidation

**DOI:** 10.1038/s41467-025-65021-6

**Published:** 2025-10-17

**Authors:** Songfeng Pei, Qinwei Wei, Kun Huang, Hui-Ming Cheng, Wencai Ren

**Affiliations:** 1 Shenyang National Laboratory for Materials Science, Institute of Metal Research, Chinese Academy of Sciences, 72 Wenhua Road, 110016 Shenyang, China; 2 School of Materials Science and Engineering, University of Science and Technology of China, 72 Wenhua Road, 110016 Shenyang, China; 3 Tsinghua-Berkeley Shenzhen Institute (TBSI), Tsinghua University, 1001 Xueyuan Road, 518055 Shenzhen, China

Correction to: *Nature Communications* 10.1038/s41467-017-02479-z, published online 10 January 2018

After concerns were raised about this publication, we informed the Journal that the original article contains an unintentional error in Fig. 3b, where the processed X-ray diffraction (XRD) spectrum of a deintercalated graphite paper sample for internal comparison was inadvertently used as the spectrum of graphite paper after 2 h intercalation (see more details in the Supporting Information below). The original source data was provided to the Journal and independently assessed by an external expert. The correct version of Fig. 3b (shown below) is updated in both the HTML and pdf version of the article. This change does not affect the results, interpretation and conclusions of the paper.
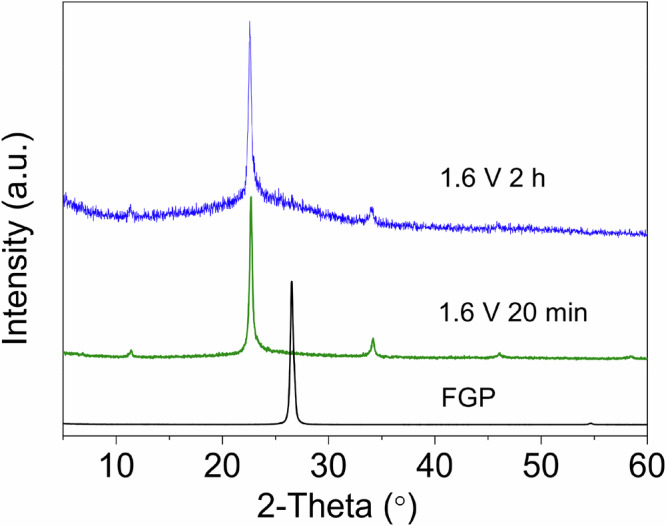


## Supplementary information

Supporting Information

