# Supplementary material for: Green synthesis of graphene oxide by seconds timescale water electrolytic oxidation
Source: Nat Commun. 2018 Jan 10;9:145. doi: 10.1038/s41467-017-02479-z (PMC5762692; doi:10.1038/s41467-017-02479-z)
Supplement: Supplementary file 1 — Supplementary Information [file 41467_2017_2479_MOESM1_ESM.pdf]

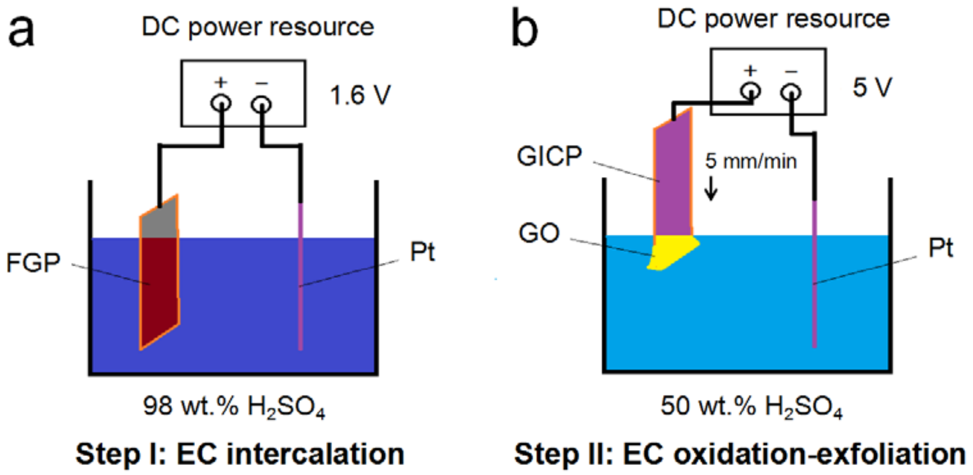

Supplementary Figure 1. Illustration of the equipment for (**a**) EC intercalation and (**b**) EC oxidization.

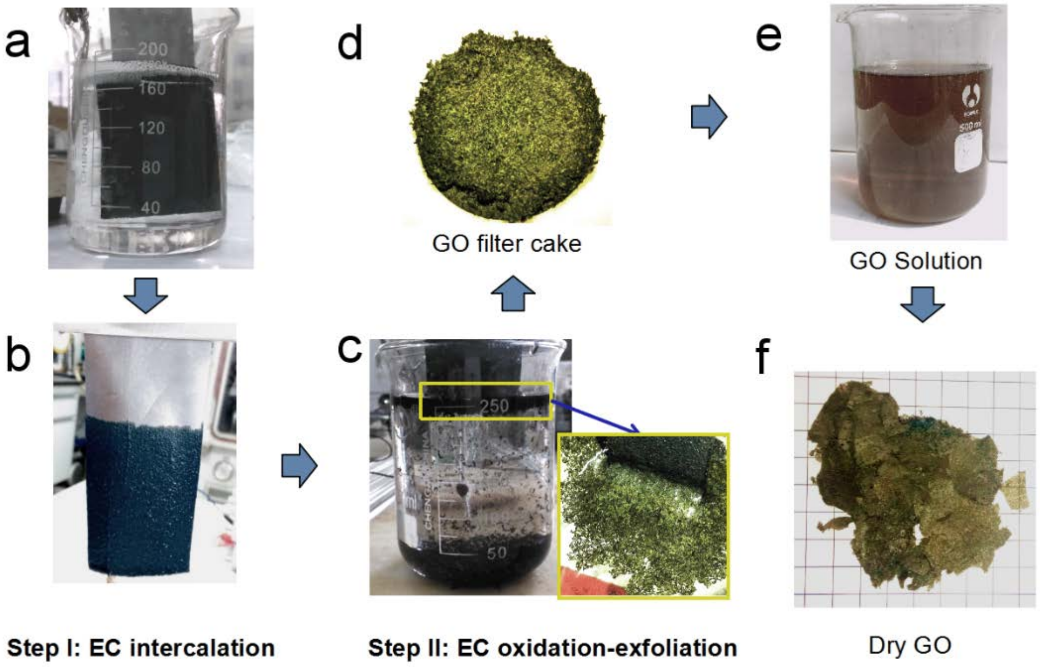

Supplementary Figure 2. Procedure of EC synthesis of EGO. a, EC intercalation of FGP in 98 wt. % H_2SO_4. **b**, Stage-I H_2SO_4-intercalated graphite compound paper (GICP). **c**, EC oxidation of GICP in 50 wt. % H_2SO_4 solution, showing that the whole GICP paper changed from blue to yellow after 3 min. **d**, A filter cake of the delaminated graphite oxide after filtration and washing. **e**, GO solution obtained by sonication. **f**, Sponge-like GO solid obtained after freezing drying.

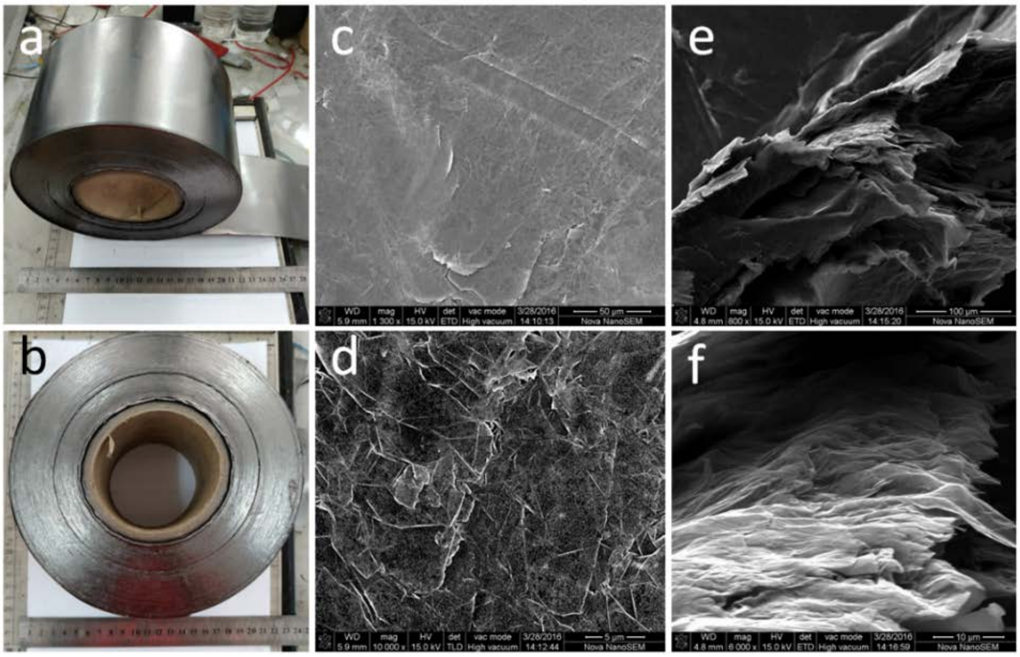

Supplementary Figure 3. Morphology of FGP. a, **b**, A roll of FGP. **c**, **d**, SEM images of the surface of FGP. **e**, **f**, SEM images of the cross section of FGP.

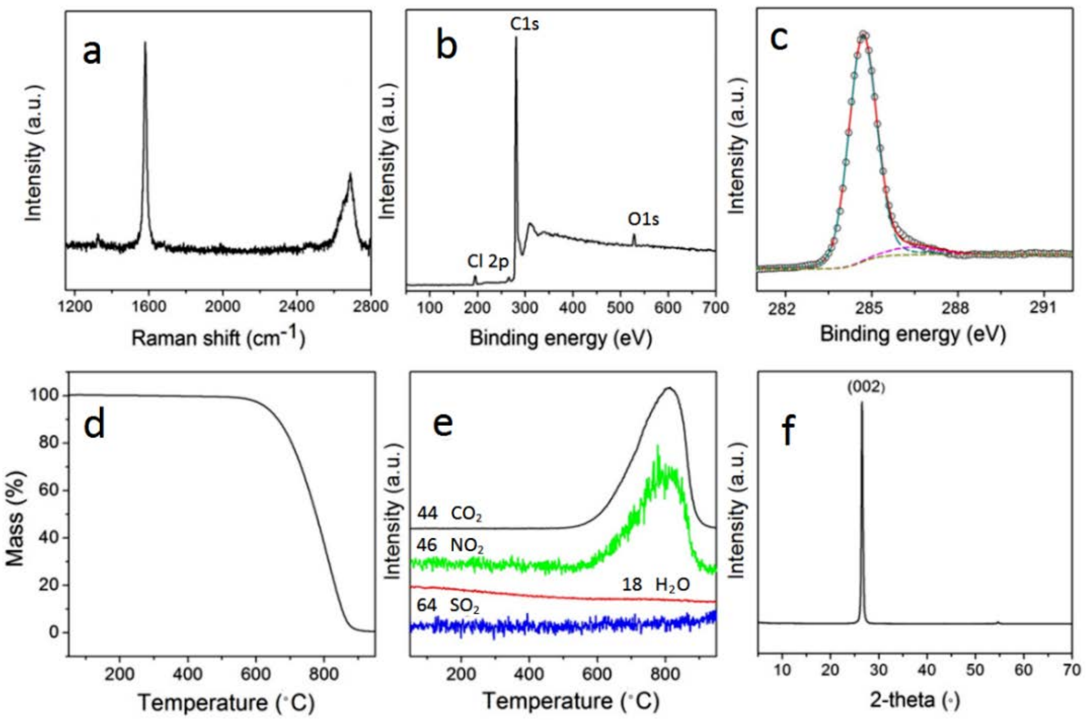

Supplementary Figure 4. Structure characterization of FGP. **a**, Raman spectrum. **b**, XPS survey. **c**, XPS C1s spectrum. **d**, TG. **e**, Thermogravimetric mass spectra (TG-MS). **f**, XRD pattern.

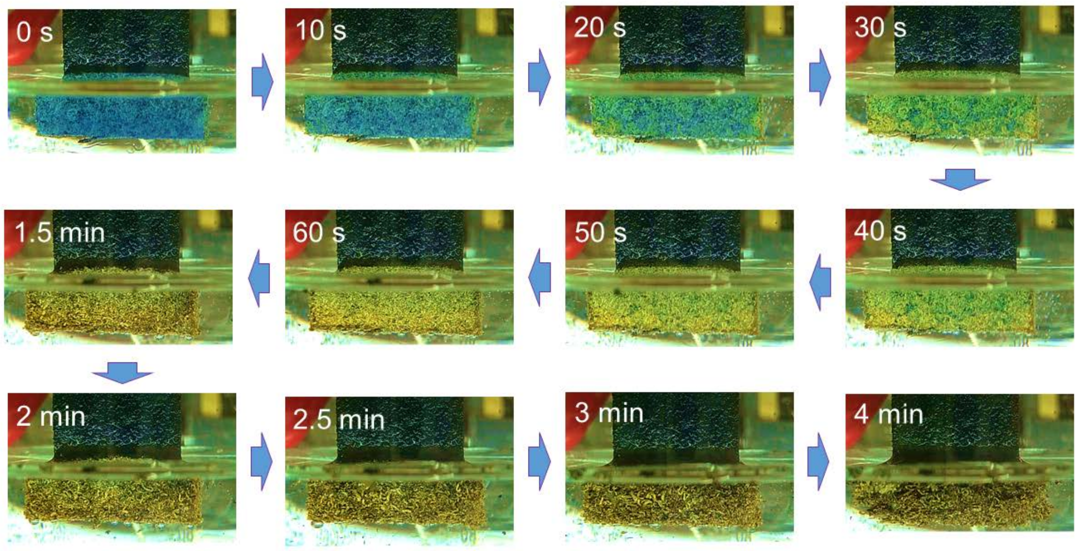

Supplementary Figure 5. Morphology and color change of a GICP slice with reaction time during static electrolytic oxidation process in 50 wt. % H_2SO_4.

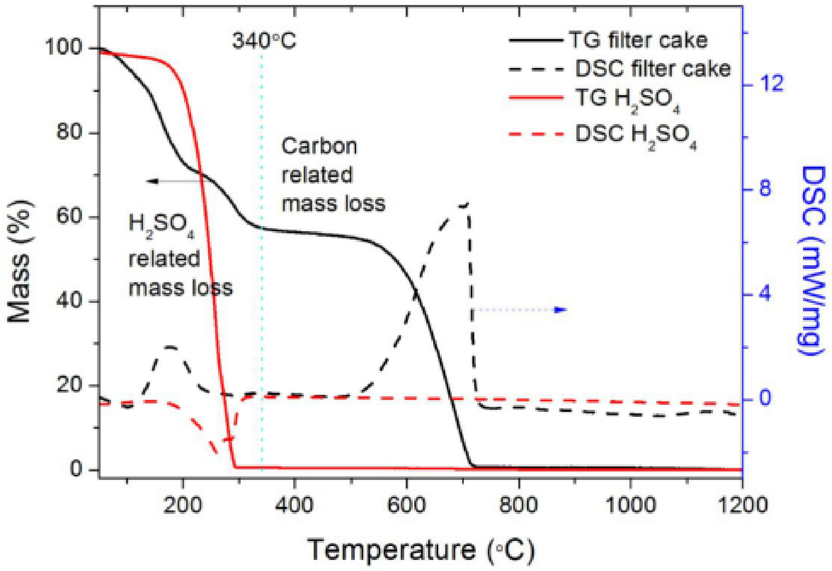

Supplementary Figure 6. TG investigation on the dried filter cake of expanded graphite oxide.

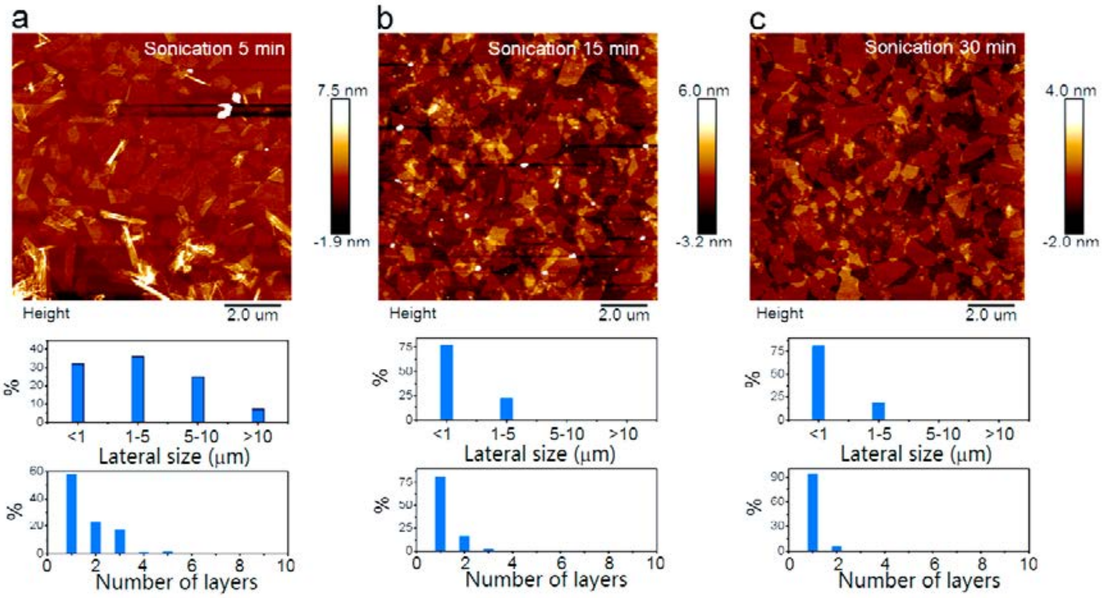

Supplementary Figure 7. Typical AFM images (top) and lateral size (middle) and thickness (bottom) distribution of EGO sheets achieved after different sonication time. **a.** 5 min, **b.** 15 min, **c.** 30 min.

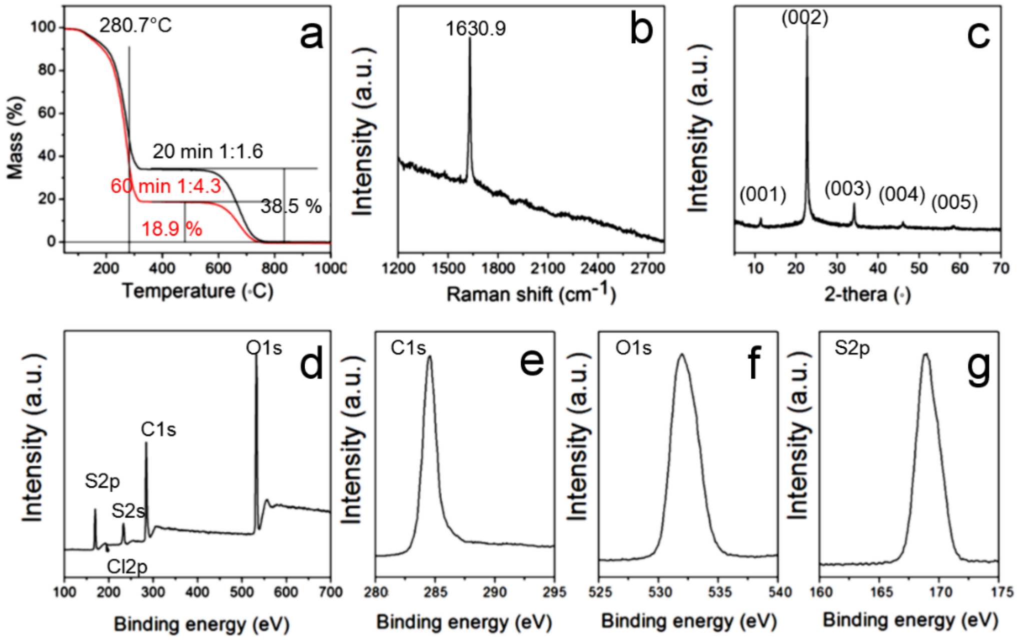

Supplementary Figure 8. Structure characterization of GICP obtained by EC intercalation at 1.2 V in 98 wt. % sulfuric acid. a, TG curves of the GICP obtained after intercalation for 20 and 60 min. **b-g**, Raman spectrum (**b**), XRD pattern (**c**), XPS survey (**d**), C1s spectrum (**e**), O1s spectrum (**f**), and S2p spectrum (**g**) of the GICP obtained after intercalation for 20 min.

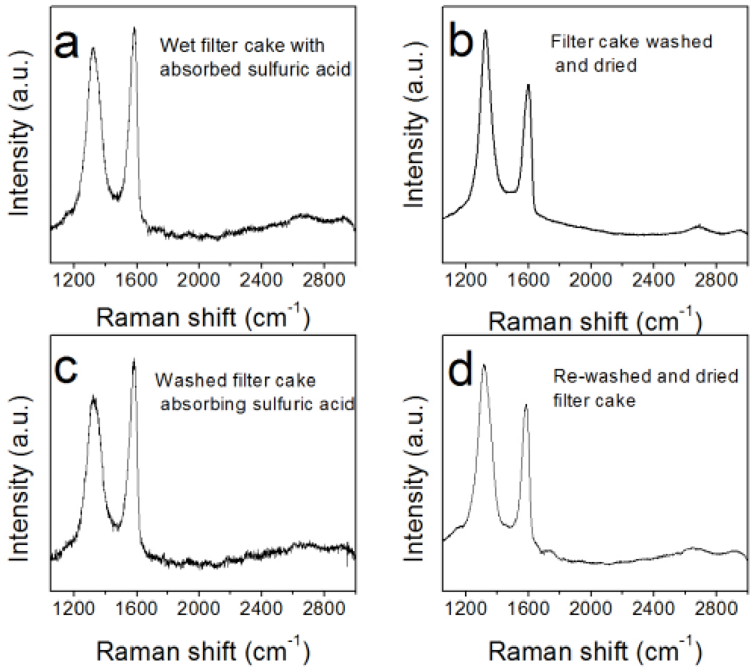

Supplementary Figure 9. Raman spectra of EGO with (**a**, **c**) and without (**b**, **d**) absorption of H_2SO_4.

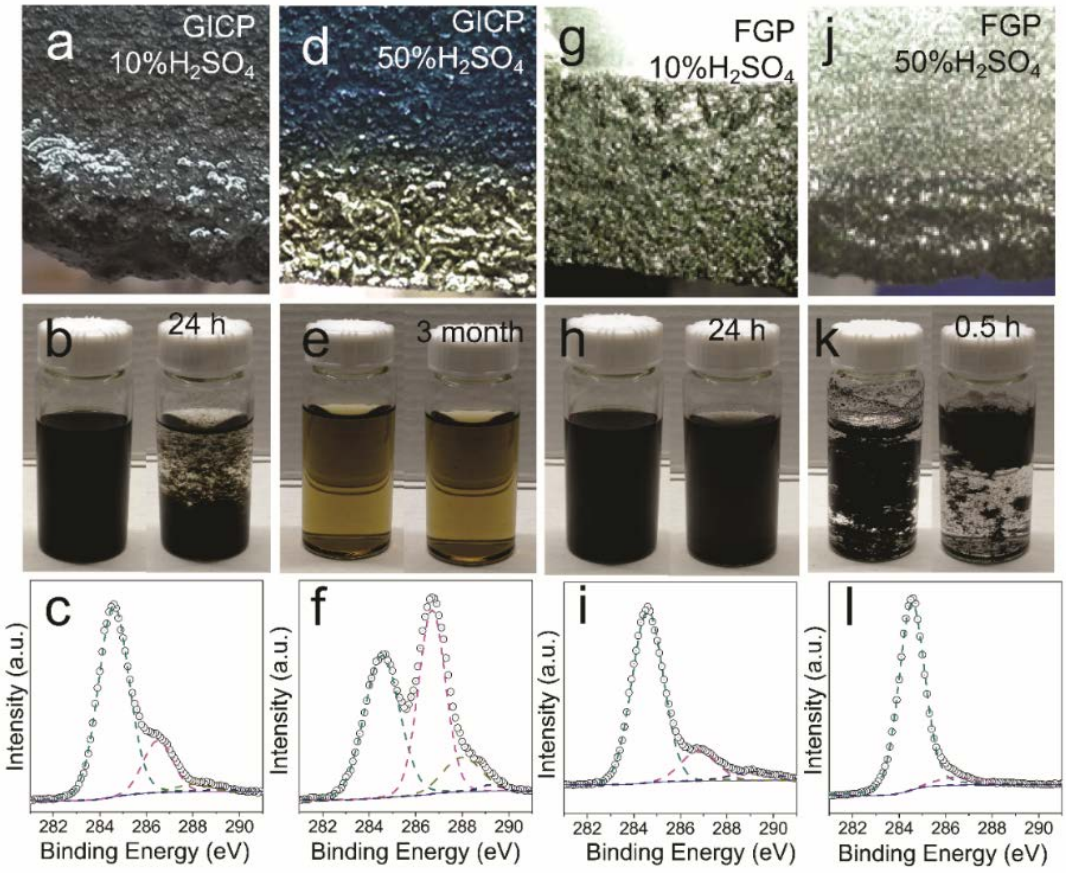

Supplementary Figure 10. Comparison on the EC oxidation and exfoliation of GICP (**a-f**) and FGP (**g-l**) in dilute sulfuric acid solution with concentration of 10 wt. % (**a-c**, **g-i**) and 50 wt. % (**d-f**, **j-l**). **a**, **d**, **g**, **j**, Optical images of GICP and FGP after reaction for 30 s. **b**, **e**, **h**, **k**, Optical images of the aqueous dispersion of exfoliated samples with an initial concentration of 1 mg·mL^{-1} before (left) and after (right) standby for different time. **c**, **f**, **i**, **l**, C1s XPS spectra of the exfoliated samples after washing and drying.

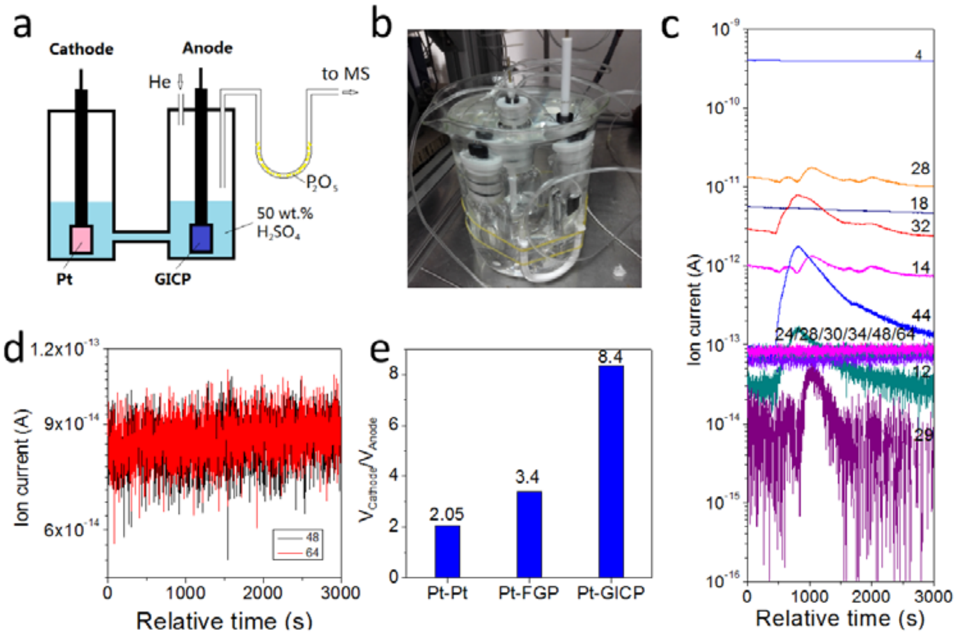

Supplementary Figure 11. **a**, **b**, Schematic (**a**) and photo (**b**) of the equipment for gas product detection. **c**, **d**, MS spectra of the gas produced by anode EC reaction during oxidation of GICP. **e**, Comparison of the volume ratio ($V_{Cathode}/V_{Anode}$) of the gases produced on a Pt cathode and different anodes (Pt/FGP/GICP) with the same EC reaction parameters.

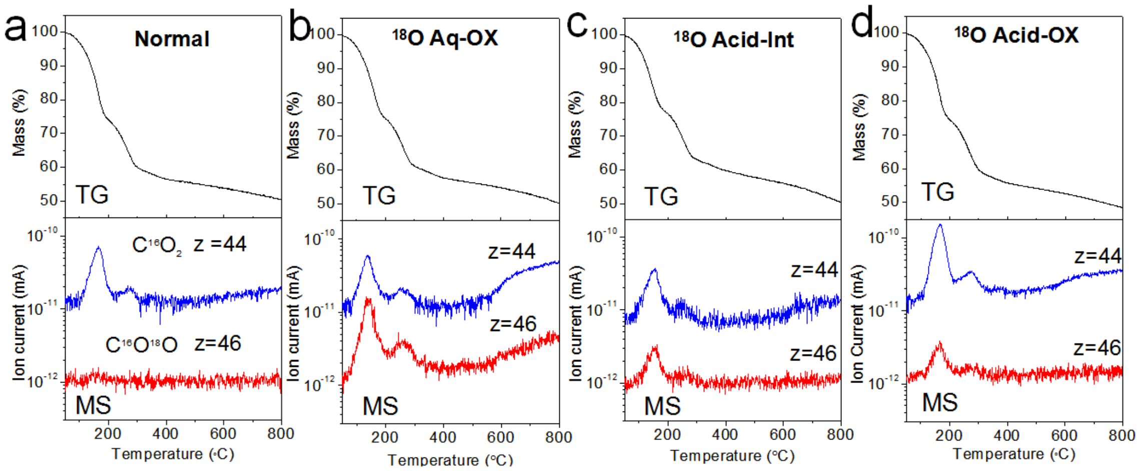

Supplementary Figure 12. TG-MS analyses of EGO synthesized from different reagent combinations. **a**, EGO synthesized with normal Acid-Int, Aq-OX, and Acid-OX (Normal). **b**, EGO synthesized with normal Acid-Int, ^{18}O Aq-OX, and normal Acid-OX (^{18}O Aq-OX). **c**, EGO synthesized with ^{18}O Acid-Int, normal Aq-OX, and normal Acid-OX (^{18}O Acid-Int). **d**, EGO synthesized with normal Acid-Int, normal Aq-OX, and ^{18}O Acid-OX (^{18}O Acid-OX).

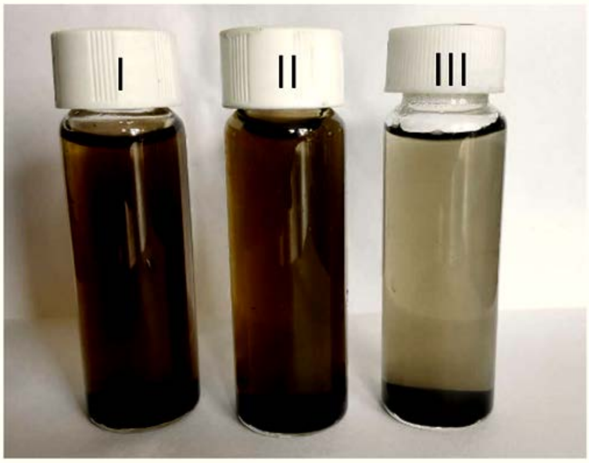

Supplementary Figure 13. Comparison on dispersion stability of the EGO samples in water (2 $mg{\cdot}mL^{-1}$), which are achieved by EC oxidation of GICP without (I) and with (II: 4 $mg{\cdot}mL^{-1}$, III: 10 $mg{\cdot}mL^{-1}$) the addition of TEMPO after standby 12 hours.

Supplementary Table 1.

Comparison of reaction parameters for GO synthesis by our water electrolytic oxidation method and traditional chemical oxidation methods.

No.	Reaction temperature	Reaction time to form graphite oxide	Ingredients consumption for 1 g graphite			C/O	Ref.
			H_2SO_4	Oxidant	Water		
**1**	~ 20 °C (Room temperature: R.T.)	a few seconds	< 0.43 mL	Highly active radicals produced by water electrolysis	< 150 g	1.5 ~ 1.8	This work
**2**	0 °C →35 °C →98 °C	> 0.75 h	23 mL	$KMnO_4$ 3 g<br>$NaNO_3$ 0.5 g	> 326 g	2.1 ~ 2.9	1
**3**	40 °C→50 °C	> 12 h	120 mL	H_3PO_4 13 mL<br>$KMnO_4$ 6 g	> 267 g	Not mentioned (N.M.)	2
**4**	80 °C→R.T. →0 °C →35 °C	> 16 h	24.5 mL	$K_2S_2O_8$ 0.5 g<br>P_2O_5 0.5 g<br>$KMnO_4$ 3 g	> 411 g	1.3	3
**5**	80 °C→R.T. →20 °C →35 °C	> 8.5 h	48 mL	$K_2S_2O_8$ 1.67 g<br>P_2O_5 1.67 g<br>$KMnO_4$ 5 g	> 317 g	N.M.	4
**6**	0 °C→ ~20 °C	124 h	59 mL	$KMnO_4$ 4.5 g<br>$NaNO_3$ 0.75 g	> 1334 g	N.M.	5
**7**	90 °C→80 °C →0 °C→35 °C	> 11.5 h	867 mL	$K_2S_2O_8$ 33.3 g<br>P_2O_5 33.3 g<br>$KMnO_4$ 400 g	91334 g	N.M.	6
**8**	R.T.	1 h	40 mL	K_2FeO_4 6 g	N.M.	2.2	7
**9**	0 °C→35 °C	> 4 h	48 mL	$KMnO_4$ 6 g<br>$NaNO_3$ 1 g	> 140 g	2.57 ~ 2.63	8
**10**	0 °C→R.T.	> 1.7 h	23 mL	$KMnO_4$ 3 g<br>$NaNO_3$ 0.5 g	> 186 g	1.7 ~ 2.5	9
**11**	0 °C→60 °C	> 5 h	23.3 mL	$KMnO_4$ 3 g<br>$NaNO_3$ 0.5 g	N.M.	1.6 ~ 2.1	10

**12**	R.T.	73 h	30 mL	$KMnO_4$ 4 g<br>$NaNO_3$ 0.75 g	N.M.	N.M.	11
**13**	0 °C→R.T.	96.5 h	17.5 mL	$KClO_3$ 11 g<br>fHNO_3 9 mL	> 800 g	2.6	12
**14**	R.T.→0 °C →35 °C→0 °C	> 2 h	25 mL	$KMnO_4$ 3.5 g	N.M.	1.23	13
**15**	80 °C→R.T.→0 °C→35 °C→55 °C	> 6.5 h	150 mL	P_2O_5 0.75 g<br>$K_2S_2O_8$ 0.75 g<br>$KMnO_4$ 5 g	> 1000 g	N.M.	14
**16**	90 °C→80 °C→R.T.→0 °C→35 °C	> 9 h	42.5 mL	P_2O_5 0.83 g<br>$K_2S_2O_8$ 0.83 g<br>$KMnO_4$ 5 g	> 1268 g	1.8	15
**17**	< 10 °C	> 27 h	25 mL	$KMnO_4$ 3 g<br>$NaNO_3$ 0.5 g	> 578 g	N.M.	16
**18**	0 °C→40 °C→95 °C	> 0.75 h	23 mL	$KMnO_4$ 3 g	> 292 g	2.36	17
**19**	R.T.	>24 h	N.M.	$NaClO_3$ 8.5 g<br>fHNO_3 20 mL	N.M.	2.8	18
**20**	80 °C→R.T.→0 °C→35 °C	>8.25 h	29 mL	$K_2S_2O_8$ 2 g<br>P_2O_5 2 g<br>$KMnO_4$ 3 g<br>$NaNO_3$ 0.5 g	> 259 g	N.M.	19
**21**	0 °C→R.T.	126 h	53.2 mL	$NaNO_3$ 0.76 g<br>$KMnO_4$ 4.5 g	1048 g	N.M.	20
**22**	0 °C→R.T.	97 h	17.5 mL	fHNO_3 9 mL<br>$KClO_3$ 11 g	> 1750 g	N.M.	21
**23**	80 °C→R.T.→0 °C→35 °C→50 °C	> 10 h	82.5 mL	$K_2S_2O_8$ 1.5 g<br>P_2O_5 1.5 g<br>$KMnO_4$ 8.75 g	1950 g	N.M.	22
**24**	R.T.→0 °C →40 °C	> 25 h	23 mL	$NaNO_3$ 0.1 g<br>$KMnO_4$ 3 g	> 186 g	N.M.	23
**25**	0 °C→R.T.	> 123 h	36 mL	fHNO_3 12 mL<br>$KMnO_4$ 5 g	1920 g	N.M.	24
**26**	0 °C→R.T.	127 h	> 78 mL	$NaNO_3$ 0.87 g<br>$KMnO_4$ 4.5 g	> 95 g	N.M.	25
**27**	80 °C→0 °C→35 °C	>8.25 h	98 mL	$K_2S_2O_8$ 2 g<br>P_2O_5 2 g<br>$KMnO_4$ 15 g	> 744 g	N.M.	26

**28**	80 °C→R.T.→35 °C	>8.5 h	44 mL	$K_2S_2O_8$ 0.83 g<br>P_2O_5 0.83 g<br>$KMnO_4$ 5 g	> 417 g	N.M.	[27]
**29**	R.T.→40 °C	>25 h	23 mL	$KMnO_4$ 0.75 g<br>$NaNO_3$ 0.1 g	> 186 g	N.M.	[28]
**30**	0 °C→R.T.	>10 h	13 mL	$NaNO_3$ 0.5 g<br>$KMnO_4$ 3 g	> 200 g	N.M.	[29]
**31**	R.T.→98 °C	170 h	> 80.5 mL	$NaNO_3$ 0.75 g<br>$KMnO_4$ 3 g	> 190 g	N.M.	[30]

Supplementary Table 2.

EC synthesis of graphene sheets.

**No.**	Raw material	Electrolyte	EC reaction condition	Characteristics of the product	Ref.
**1**	Natural graphite flake or HOPG	100 mL 2.25 wt. % H_2SO_4 aqueous solution added with 11 mL of 30 wt. % KOH aqueous solution; pH: ~1.2	Step 1: +2.5 V, 1 min<br>Step 2: alternating between +10 V (2 s) and -10 V (5 s)	Yield: ~ 5 to 8 wt. %;<br>Thickness: < 3 nm, 65 % < 2 nm;<br>Size: 1 to 40 μm;<br>Mobility: 5.5 – 17 $cm^2 \cdot V^{-1}s^{-1}$;<br>TCF: 43.2 $k\Omega \cdot \square^{-1}$ @ 96 % transparency	31
**2**	HOPG or graphite powders	30 $mg \cdot mL^{-1}$ solution of $LiClO_4$ in propylene carbonate (PC)	HOPG (50 mg) was used as the negative electrode and electrochemically charged at a voltage of 15 ± 5 V in electrolyte	Yield: > 70 %;<br>Sheet resistance of the film with 0.7 $mg \cdot cm^{-2}$ graphene: 15 $\Omega \cdot \square^{-1}$	32
**3**	Natural graphite flakes adhered on a conductive carbon tape	0.1 M H_2SO_4 aqueous solution	DC +10 V, 2 min	Yield: > 80 % of 1 – 3 layers;<br>C/O: 12.3;<br>Single sheet resistance: 4.8 $k\Omega \cdot \square^{-1}$	33
**4**	Natural graphite flakes adhered on carbon tape	Aqueous solutions of $(NH_4)_2SO_4$, Na_2SO_4, K_2SO_4, etc.;<br>Concentration: 0.1 M; pH: ~6.5 − 7.0	DC +10 V, 3 − 5 min	Yield (> 85 %, ≤ 3 layers);<br>Lateral size: up to 44 μm;<br>C/O: 17.2;<br>Hole mobility: 310 $cm^2 \cdot V^{-1}s^{-1}$	34
**5**	Graphite foil	Step 1: +10 V, 10 min, 1 M NaOH aqueous solution<br>Step 2: +10 V, 50 min, 0.5 M H_2SO_4 aqueous solution		Yield: > 56 %;<br>I_D/I_G: < 0.29;<br>C/O: ~ 11.02	35

**6**	Graphite foil (pretreatment by freezing and thawing cycles)	0.1 M $(NH_4)_2SO_4$ aqueous solution with addition of 0.1 % TEMPO	DC +10V, <10 s	Lateral size: 5 – 10 µm;<br>C/O: 25.3;<br>Hole mobility: ~ 405 $cm^2 \cdot V^{-1}s^{-1}$;<br>I_D/I_G: < 0.1	36

Supplementary Table 3.

EC synthesis of partially oxidized graphene sheets.

No.	Raw material	Electrolyte	EC reaction condition	Characteristics of the product	Ref.
**1**	Pencil cores	Aqueous electrolyte of H_2SO_4 or H_3PO_4	Step 1: +1 V, 3 – 5 min;<br>Step 2: +7 V, 5 – 8 min and alternation between +7 V and -7 V every 5–8 min, repeatedly	I_D/I_G: 0.71;<br>Thickness: 1 – 5 atomic layers, mostly 3 – 12 nm;<br>XRD main peak: 26.4° (characteristic of graphite rather than graphite oxide)	37
**2**	Expanded graphite flakes pressed into a pellet	10 M H_2SO_4 aqueous solution	Step 1: +1 V, 10 min;<br>Step 2: +2 V, 20 min	Yield: ~ 80 %;<br>I_D/I_G: < 0.3;<br>C/O: ~ 6.8	38
**3**	Graphite rods	1 M $(NH_4)_2SO_4$ aqueous solution	Constant current of 1.0 A for 2 h	Yield: > 80 wt. %;<br>Thickness: 1 – 4 layers, 80 %;<br>I_D/I_G: 0.85;<br>Sheet resistance: 1.5 $k\Omega\cdot\square^{-1}$ (electrical conductive);<br>The oxygen functional groups are decorated dominantly at the edges, and the basal plane is nearly intact	39
**4**	Graphite flakes	1 M H_2SO_4 in saturated $(NH_4)_2SO_4$ aqueous solution	Graphite flakes in electrolyte contact anode by stirring;<br>Constant current 0.6 A for 48 h	Yield: 38.8 %;<br>C/O: ~ 3.64;<br>Lower concentration of C=O groups, lower thermal stability and dispersion stability in water compared to the fully oxidized HGO;<br>Thickness: monolayer, ~ 66 %	40
**5**	Graphite	8 M	Linear sweep	C/O: ~ 9.81	41

	flakes closed in platinum mesh	Perchloric acid ($HClO_4$) aqueous solution	voltammetry measurement was performed to the graphite electrode from ~0.2 V to 1.4 V with scan rate of 0.01 mV·s^{-1} (total time ~33 h)		
**6**	Graphite pellet obtained by pressing graphite flakes	11.6 M $HClO_4$ aqueous solution	Graphite pellet as anode and Pt plate as cathode separated by glass fiber filter membrane;<br>Constant current density *i* = 50 μA·mg^{-1} for 48 h	C/O: ~ 5.7 (~ 2 h reaction);<br>C/O: ~ 3.3 (~ 17 h reaction);<br>C/O: ~ 3.0 (~ 48 h reaction);<br>Thickness: 1 – 2 nm, ~36 %	42

Supplementary Table 4.

Comparison on the properties of GO sheets synthesized by modified Hummers method using FGP and graphite flakes as raw material.

Raw material		Reagents			Properties of GO	
		$NaNO_3$ (g)	$KMnO_4$ (g)	H_2SO_4 (mL)	C/O	zeta potential (mV)
Graphite flakes (0.5 g)	mean particle size of ~150 μm	0.5	2.5	25	2.07	-45.7
FGP (0.5 g)	Slice with thickness of 0.5 mm and lateral size of 2 mm × 2 mm	0.5	2.5	25	2.84	-17.2

Supplementary Table 5.

Comparison on the properties of FGP before and after intercalation.

Material (synthesis condition)	Color	Thickness	Fracture force	Surface resistance
		mm	N	$\Omega\cdot\square^{-1}$
FGP	Lustering-grey	0.52	3.5	0.8
GICP (1.6 V, 20 min)	Deep blue	2.03	3.3	0.3
GICP (1.6 V, 60 min)	Deep blue	3.45	-*	1.2

Note: * These properties cannot be measured because of severe swelling.

Supplementary Table 6.

Chemical composition and exfoliation rate (r_{exfo}) of the samples synthesized with different reaction conditions.

Reaction condition		Chemical composition					r_{exfo}
Anode material	$C_{electrolyte}$	C=C	C-O	C=O	O-C=O	C/O	$mm \cdot min^{-1}$
GICP	10 %	76.78 %	19.29 %	3.04 %	0.89 %	5.7	8.3
GICP	50 %	44.75 %	42.94 %	11.14 %	1.17 %	1.7	4.6
FGP	10 %	78.06 %	15.39 %	0.47 %	6.08 %	7.8	2.8
FGP	50 %	94.22 %	5.43 %	<0.01 %	0.34 %	11.5	9.7

Supplementary Table 7.

Standard mass spectrometry of the substances that are possibly present in the gaseous product of anodic EC reaction for GICP oxidation.

Substance	Mass spectrometry (*z*)
He	**4**[*]
CO	12[†], 14, 16, **28**, 29, 30
O_2	16, **32**, 33, 34
CO_2	12, 13, 16, 22, 28, 29, **44**, 45, 46
SO_2	16, 24, 32, 34, 48, 49, 50, **64**, 65, 66

Note: [*] The underlined bold number represents the maximum abundance value.
[†] The underlined number represents the second maximum abundance value.

Supplementary Table 8.

Chemical composition of the EGO samples synthesized without and with the addition of TEMPO.

Samples	TEMPO concentration in electrolyte	Elemental composition (at. %)				C/O
		C	O	H	S	
I	0	50.1	28.5	20.4	1.0	1.76
II	4‰	55.1	24.8	19.2	0.9	2.22
III	10‰	57.6	22.5	18.8	1.1	2.56

Supplementary Note 1: Characterization of FGP

The morphology and structure of the FGP used are shown in Supplementary Figs. 3 and 4. FGP is an industrial product of natural graphite, which is produced by roller-pressing expanded graphite flakes (EGFs) without use of any binder. It usually has a thickness from micrometers to millimeters, width up to meters, and length up to kilometers (Supplementary Figs. 3a and b). As shown in Supplementary Figs. 3c-f, the EGFs are interlocked together in the FGP. As shown in Supplementary Fig. 4, FGP shows similar Raman, XPS, TG, MS spectra and XRD pattern with graphite, indicating their similar structure. As a result, FGP has good flexibility, good mechanical properties with tensile strength of 4 to 5 MPa, and excellent electrical conductivity comparable with HOPG. However, the price of FGP is much cheaper than that of HOPG, which is no more than 3 times that of natural graphite flakes (32 mesh). Therefore, FGP is an ideal raw material for EC synthesis of graphene and GO especially to achieve continuous and automatic mass production because of its continuous, flexible, strong and conductive characteristics.

Supplementary Note 2: Estimation on the absorption quantity of H_2SO_4 in graphite oxide cake and the water consumption for cleaning

The wet filter cake of delaminated graphite oxide contains sulfuric acid and water, and its composition was determined as following:

(1) The wet filter cake was first dried at 60°C for more than 24 h, leaving ~8.5 wt.% solid matter. This means ~91.5 wt.% matter in the filter cake is water since most of H_2SO_4 cannot evaporate with water by mild heating.

(2) Then the dried filter cake was tested by TG, and the result is shown in Supplementary Fig. 6. The mass loss of ~ 44 wt.% before 340 °C is related to the evaporation of sulfuric acid, while the residue (~56 wt.%) is carbon related materials.

It is worth noting that the sulfuric acid solution absorbed in the wet cake has a very low H_2SO_4 concentration of no more than 4.0 wt.% ($\frac{8.5\times0.44}{91.5+(8.5\times0.44)} \approx 3.7\ \%$), although the concentration of the H_2SO_4 solution used for oxidation reaction is 50 wt.%. This result indicates that the delaminated graphite oxide is more likely to absorb water rather than H_2SO_4 or related ions, and therefore they are much easier to be cleaned by water washing.

We then measured the amount of water used to clean graphite oxide prepared by our EC oxidation method. The criterion for full purification is that the solution after cleaning has a conductivity less than 50 $\mu S\cdot cm^{-1}$ and pH higher than 4. It was found that no more than 150 g of distilled water is enough for the purification of 1 g of EGO. In contrast, more than 1.5 kg of distilled water is needed for the purification of

1 g of GO prepared by Hummers method to reach the same criterion (see Supplementary Table 1).

Supplementary Note 3: Characterization of GICP

The GICP synthesized by EC intercalation at 1.6 V for 20 min was characterized by TG (in air), Raman spectroscopy, XRD, and XPS (Supplementary Fig. 8).

As shown in Supplementary Fig. 8a, the TG curve shows a sharp mass loss around ~280 ºC, which is mainly caused by the volatilization or decomposition of sulfuric acid that is intercalated into graphite gallery. Based on this data, we estimate that the weight ratios of host graphite to the intercalant are 1:1.6 and 1:4.3 for intercalation of 20 and 60 min, respectively.

Supplementary Fig. 8b shows the Raman spectrum of GICP in the range of 1200 cm^{-1} to 2700 cm^{-1}. The absence of obvious D peak indicates that no defect was generated during the intercalation process. The G peak at 1631 cm^{-1} is consistent with that of the stage-I H_2SO_4-GIC. [43, 44] The 2D band is obviously suppressed by the "Pauli blocking" effect of intercalation. [43, 45, 46]

XRD was also used to investigate the structure of GICP. As shown in Supplementary Fig. 8c, the GICP achieved by intercalation for 20 min shows the XRD characteristic of stage-I GICP. [47] The interlayer distance was calculated to be 7.79 Å ($2\theta_{(001)}$ = 11.35º) based on the Bragg equation.

XPS was used to investigate the chemical structure of the surface of GICP. The samples were used as-synthesized without washing with water. Due to the absorbed sulfuric acid in GICP, both O1s and S2p peaks are observed (Supplementary Figs. 8d, f, g). However, the C1s spectrum shows the same feature as that of graphite

(Supplementary Fig. 8e), indicating that no oxidation and new chemical bonds were formed during intercalation.

The macroscopic properties of FGP and GICP are shown in Supplementary Table 5. After intercalation, the color of FGP changes from lustering-grey to deep blue. Compared to FGP, the GICP obtained after intercalation at 1.6 V for 20 min shows 4 times increase in thickness, but nearly the same fracture force and good flexibility. In addition, the surface resistance decreases from 0.8 for FGP to 0.3 $\Omega\cdot\square^{-1}$ for GICP because of the doping effect of sulfuric acid. Intercalation for longer time (60 min) leads to severe swelling, a very low mechanical strength that could not be measured and an increase in surface resistance to 1.2 $\Omega\cdot\square^{-1}$.

Supplementary Note 4: Raman spectra of EGO with and without absorption of H_2SO_4

In our experiments, we found that the I_D/I_G of EGO obtained during in-situ Raman investigations (I_D/I_G<1, Fig. 3d in main text) is smaller than that of the final EGO product (I_D/I_G >1, Fig. 2d in main text) although the EC reaction time is the same. To understand this difference, a newly as-synthesized EGO sample collected by filtration without washing and drying was measured. It can be seen that the Raman spectrum (Supplementary Fig. 9a) is similar to that obtained during in-situ Raman measurements. Interestingly, after the EGO sample was washed and dried to remove the absorbed water and H_2SO_4, it shows a similar Raman spectrum with the final EGO product (Supplementary Fig. 9b). To confirm such change, the dried clean EGO sample was re-wetted with H_2SO_4 solution and then re-washed and dried again for Raman measurements separately. As shown in Supplementary Figs. 9c and d, they show the same Raman spectra as the original wet and dried samples. These results indicate that the smaller I_D/I_G observed in our in-situ Raman measurements is attributed to the water and H_2SO_4 contained in the as-synthesized EGO samples.

Supplementary Note 5. EC exfoliation and oxidation of GICP and FGP in H_2SO_4 solutions with different concentrations

The exfoliation of GICP and FGP in H_2SO_4 solution is much different from each other. As samples, we studied the EC exfoliation of GICP and FGP in dilute sulfuric acid solutions with concentrations of 10 wt. % and 50 wt. %, and the results are shown in Supplementary Fig. 10 and Supplementary Table 6. The GICP slice in 10 wt. % solution and the FGP slice in 50 wt. % solution quickly swelled, expanded and exfoliated once dipping into the electrolyte, while the GICP slice in 50 wt. % solution and the FGP slice in 10 wt. % solution showed no obvious exfoliation. For the latter case, the surface of FGP slice changed from smooth-lustering-gray to wrinkled-deep-gray, while the GICP slice immersed in the liquid changed color from deep-blue to yellow-brown along with the appearance of worm-like particles, which are the sign of the formation and expansion of graphite oxide. The gradual morphology and color changes of GICP during EC oxidation in 50 wt. % H_2SO_4 solution are shown in Supplementary Fig. 5 and Supplementary Movie 3. As summarized in Supplementary Table 6, the chemical compositions of the products formed in the four conditions are different from each other. The oxidation degrees of the products with GICP as anode are generally higher than that with FGP as anode. The higher oxidation degree, the more stable dispersion of exfoliated product. Moreover, the high oxidation degree is correlated with low exfoliation rate. These results indicate that both pre-intercalation of FGP by H_2SO_4 and the concentration of

H_2SO_4 in electrolyte are essentially important for ultrafast synthesis of fully oxidized GO sheets.

Supplementary Note 6. Gaseous product analysis of EC reactions with different anode and cathode

In order to determine the products of anodic EC reaction for GICP oxidation, the gaseous product at anode was collected by a home-made equipment as shown in Supplementary Figs. 11a and b, and measured by an on-line mass spectrometer (Catlab QIC20, Hiden Analytical) with helium (He) gas as carrier. Before EC reaction, the air in the valve containing GICP anode was fully replaced by He gas. According to the results shown in Supplementary Figs. 11c, d and the standard mass spectrometry of the substances that are possibly present in the gaseous product in Supplementary Table 7, the gaseous product contains only O_2, CO, and CO_2. It is worth noting that no SO_2 was detected, indicating that H_2SO_4 was not decomposed at anode during EC oxidation process of GICP. This is consistent with the results that were obtained from the ^{18}O isotropic tracing experiments shown below.

The gases produced on both electrodes were collected and their volumes were measured with eudiometers. Since the mixed gas could not be ignited by electric sparking and there was no obvious volume change after ignition, we considered that the gaseous product collected from the GICP anode contains only a trace amount of CO. After ignition and alkali washing to remove CO and CO_2, only O_2 was left, which takes ~96 % of the original volume. These results indicate that a small amount of carbon in GICP was oxidized into CO_2 or CO during EC reaction as reported previously [48].

For Pt cathode and different anodes (Pt/FGP/GICP), the volume ratios of the gases produced on cathode (H_2) and anode (mainly O_2) with the same EC reaction parameters are shown in Supplementary Fig. 11e. The gas ratio obtained by Pt cathode and anode is 2.05, approaching the theoretical value of H_2 and O_2 produced by water electrolysis (2.0). The main electrode reactions are shown as below:

Pt Cathode: $4H^+ + 4e^- = 2H_2\uparrow$

Pt Anode: $2H_2O = O_2\uparrow + 4H^+ + 4e^-$

The gas ratio from the Pt cathode and FGP anode is 3.4. The low O_2 production is because the anode reaction also contains intercalation reaction of HSO_4^- in addition to water electrolysis as shown below:

Pt Cathode: $4H^+ + 4e^- = 2H_2\uparrow$

FGP Anode: $2H_2O = O_2\uparrow + 4H^+ + 4e^-$

$G + nHSO_4^- = GIC + ne^-$

The gas ratio from the Pt cathode and GICP anode is 8.4. Since there is no further intercalation reaction, the high gas ratio indicates that the formation of O_2 is greatly inhibited. As a result, the sufficient adsorbed reactive *OH, *O and *OOH together with the high current density enable the ultrafast synthesis of fully oxidized graphite oxide by reacting with the carbon lattice that has been highly positively charged. The main electrode reactions are shown as below:

Pt Cathode: $4H^+ + 4e^- = 2H_2\uparrow$

GICP Anode: $G + nH_2O = nH^+ + G(OH)_n + ne^-$

$2H_2O = O_2\uparrow + 4H^+ + 4e^-$

Supplementary Note 7. Isotopic tracing experiments on the oxygen source of EGO

In our method, the reagents used for EGO synthesis can be divided into three types: concentrated H_2SO_4 used for EC intercalation (Acid-Int), water (Aq-OX) and H_2SO_4 (Acid-OX) in electrolyte for EC oxidation. To trace the transfer path of oxygen, we replaced the three type reagents by their isotopic homologs containing ^{18}O, $H_2{}^{18}O$ and $H_2S^{16}O_3{}^{18}O$, separately for EGO synthesis. Four different EGO samples were synthesized with different combinations of reagents. The ^{18}O content of each EGO product was measured by TG-MS with Netzsch STA 449C Jupiter/QMS 403C using Helium (He) as carrier and the results are shown in Supplementary Fig. 12.

The TG curves of the four EGO samples are similar with each other. In inert atmosphere (He), the main mass loss of ~40 wt. % occurs below 300 °C, and the full mass loss of ~50 wt. % till 800 °C, which are consistent with the feature of TG curve of GO synthesized by the Hummers' method. In our experiments, the most detectable substance in MS is carbon dioxide (CO_2), which is the pyrolysis product of GO and EGO. We used the mass-to-charge ratios of $C^{16}O_2$ (z_{44}) and its isotopic homolog $C^{16}O^{18}O$ (z_{46}) to evaluate the content of ^{18}O in the sample. We calculated the peak areas of z_{46} and z_{44} curves in each TG-MS spectrum from 100 ºC to 300 ºC with a linear background, and the peak area ratio z_{46}/z_{44} was used to evaluate the ^{18}O content in the four samples.

As shown in Supplementary Fig. 12a, without the addition of ^{18}O-containing reagent, the z_{46}/z_{44} of EGO sample (Normal) is only ~0.05 %. When the water in

electrolyte used in the oxidation process was replaced by $H_2{}^{18}O$ (97 %), the z_{46}/z_{44} of EGO sample (^{18}O Aq-OX) is ~35.1 % (Supplementary Fig. 12b). When the H_2SO_4 used for intercalation was replaced by $H_2S^{16}O_3{}^{18}O$, the z_{46}/z_{44} of EGO sample (^{18}O Acid-Int) is ~5.0 % (Supplementary Fig. 12c). When the H_2SO_4 in electrolyte used in the oxidation process was replaced by $H_2S^{16}O_3{}^{18}O$, the z_{46}/z_{44} of EGO sample (^{18}O Acid-OX) is ~1.8 % (Supplementary Fig. 12d). These results are summarized in Fig. 3i in main text, which clearly show that water in the electrolyte is the dominant source for the oxygen functional groups in our EGO.

Supplementary Note 8. Evidence on the presence of oxygen radical intermediates and their effect on the oxidation of graphite during EC oxidation process

It has been reported that 2,2,6,6-tetramethylpiperidin-1-oxyl (TEMPO) can efficiently suppress the formation of oxygen radicals from water electrolysis[36]. We studied the influence of addition of TEMPO on the synthesis of EGO sheets under the optimized EC oxidation conditions of GICP (~50 wt. % H_2SO_4 aqueous solution, 5V). All the as-synthesized products were exfoliated in water to form suspensions with a concentration ~2 mg·mL^{-1}. As shown in Supplementary Fig. 13, after standby 12 hours, the EGO products achieved without (sample I) and with the addition of a small amount (4‰, sample II) of TEMPO show good stability with no obviously sediment, while the product achieved with the addition of a relatively large amount of TEMPO (10‰, sample III) totally precipitates. Elemental analyses show that adding 4‰ and 10‰ of TEMPO leads to a increase in C/O ratio of the products from ~1.8 to ~2.2 and ~2.6, respectively (Supplementary Table 8). This gives strong evidence of the existing of oxygen radicals and their key role in the synthesis of GO during our EC oxidation process.

Supplementary Note 9. Recycling of sulfuric acid

There are two sources for the sulfuric acid that need to be recycled in our method.

After intercalation, besides the intercalated HSO_4^- and H_2SO_4, there is still a large amount of sulfuric acid absorbed on the surface of or inside the GICP, leading to 5 to 6 times mass increase for FGP. This is the first source. In our experiments, a pressing step was used to remove the absorbed sulfuric acid, which was then recycled into the intercalation bath. After pressing, the mass ratio of graphite to sulfuric acid in GICP was estimated to be around 1:1.6 based on TG measurements (Supplementary Fig. 8a). This value is still a little bit larger than the theoretical value (~1:1.02) for the stage-I H_2SO_4 intercalated GICP, indicating the presence of a small amount of absorbed H_2SO_4. This absorbed and intercalated sulfuric acid will dissolve into the sulfuric acid solution used for EC oxidation in the second step.

The very dilute H_2SO_4 solution obtained after cleaning graphite oxide is another source. Note that there is a small amount of loss of water during EC oxidation process because of evaporation and electrolysis, which leads to a small increase in the concentration of H_2SO_4 in the electrolyte. Therefore, the very dilute H_2SO_4 solution was added into the electrolyte after a certain time of EC oxidation to recover to its original concentration. Moreover, it can also be used for the production of concentrated H_2SO_4 by absorbing SO_3.

Therefore, the sulfuric acid used in our methods can be fully recycled.

Supplementary References

1. Hummers, W.S. & Offeman, R.E. Preparation of graphitic oxide. *J. Am. Chem. Soc.* **80**, 1339-1339 (1958).
2. Marcano, D.C. et al. Improved synthesis of graphene oxide. *ACS Nano* **4**, 4806-4814 (2010).
3. Kovtyukhova, N.I. et al. Layer-by-layer assembly of ultrathin composite films from micron-sized graphite oxide sheets and polycations. *Chem. Mater.* **11**, 771-778 (1999).
4. Zhou, X.Z. et al. In situ synthesis of metal nanoparticles on single-layer graphene oxide and reduced graphene oxide surfaces. *J. Phys. Chem. C* **113**, 10842-10846 (2009).
5. Hirata, M., Gotou, T., Horiuchi, S., Fujiwara, M. & Ohba, M. Thin-film particles of graphite oxide 1: High-yield synthesis and flexibility of the particles. *Carbon* **42**, 2929-2937 (2004).
6. Luo, Z., Lu, Y., Somers, L.A. & Johnson, A.T.C. High yield preparation of macroscopic graphene oxide membranes. *J. Am. Chem. Soc.* **131**, 898-899 (2009).
7. Peng, L. et al. An iron-based green approach to 1-h production of single-layer graphene oxide. *Nat. Comm.* **6**, 5716 (2015).
8. Zhao, J., Pei, S., Ren, W., Gao, L. & Cheng, H.-M. Efficient preparation of large-area graphene oxide sheets for transparent conductive films. *ACS Nano* **4**, 5245-5252 (2010).

9. Wu, Z.S. et al. Synthesis of high-quality graphene with a pre-determined number of layers. *Carbon* **47**, 493-499 (2009).

10. Tölle, F.J., Gamp, K. & Mülhaupt, R. Scale-up and purification of graphite oxide as intermediate for functionalized graphene. *Carbon* **75**, 432-442 (2014).

11. Su, Q. et al. Composites of graphene with large aromatic molecules. *Adv. Mater.* **21**, 3191-3195 (2009).

12. Schniepp, H.C. et al. Functionalized single graphene sheets derived from splitting graphite oxide. *J. Phys. Chem. B* **110**, 8535-8539 (2006).

13. Park, S. et al. Aqueous suspension and characterization of chemically modified graphene sheets. *Chem. Mater.* **20**, 6592-6594 (2008).

14. Luo, J.Y. et al. Graphene oxide nanocolloids. *J. Am. Chem. Soc.* **132**, 17667-17669 (2010).

15. Gilje, S., Han, S., Wang, M., Wang, K.L. & Kaner, R.B. A chemical route to graphene for device applications. *Nano Lett.* **7**, 3394-3398 (2007).

16. Eigler, S. et al. Wet chemical synthesis of graphene. *Adv. Mater.* **25**, 3583-3587 (2013).

17. Chen, J., Yao, B., Li, C. & Shi, G. An improved hummers method for eco-friendly synthesis of graphene oxide. *Carbon* **64**, 225-229 (2013).

18. Shin, H.-J. et al. Efficient reduction of graphite oxide by sodium borohydride and its effect on electrical conductance. *Adv. Funct. Mater.* **19**, 1987-1992 (2009).

19. He, S.J. et al. A graphene nanoprobe for rapid, sensitive, and multicolor fluorescent DNA analysis. *Adv. Funct. Mater.* **20**, 453-459 (2010).

20. Eda, G., Fanchini, G. & Chhowalla, M. Large-area ultrathin films of reduced graphene oxide as a transparent and flexible electronic material. *Nat. Nanotech.* **3**, 270-274 (2008).

21. Lomeda, J.R., Doyle, C.D., Kosynkin, D.V., Hwang, W.F. & Tour, J.M. Diazonium functionalization of surfactant-wrapped chemically converted graphene sheets. *J. Am. Chem. Soc.* **130**, 16201-16206 (2008).

22. Tang, L.H. et al. Preparation, structure, and electrochemical properties of reduced graphene sheet films. *Adv. Funct. Mater.* **19**, 2782-2789 (2009).

23. Wang, H.L., Robinson, J.T., Li, X.L. & Dai, H.J. Solvothermal reduction of chemically exfoliated graphene sheets. *J. Am. Chem. Soc.* **131**, 9910-9911 (2009).

24. Shao, Y.Y., Wang, J., Engelhard, M., Wang, C.M. & Lin, Y.H. Facile and controllable electrochemical reduction of graphene oxide and its applications. *J. Mater. Chem.* **20**, 743-748 (2010).

25. Ji, L. et al. Graphene oxide as a sulfur immobilizer in high performance lithium/sulfur cells. *J. Am. Chem. Soc.* **133**, 18522-18525 (2011).

26. Liu, S.B. et al. Antibacterial activity of graphite, graphite oxide, graphene oxide, and reduced graphene oxide: membrane and oxidative stress. *ACS Nano* **5**, 6971-6980 (2011).

27. Yang, S.T. et al. Folding/aggregation of graphene oxide and its application in Cu^{2+} removal. *J. Colloid & Interface Sci.* **351**, 122-127 (2010).

28. Li, X.L. et al. Simultaneous nitrogen doping and reduction of graphene oxide. *J. Am. Chem. Soc.* **131**, 15939-15944 (2009).

29. Liu, C.G., Yu, Z.N., Neff, D., Zhamu, A. & Jang, B.Z. Graphene-based supercapacitor with an ultrahigh energy density. *Nano Lett.* **10**, 4863-4868 (2010).

30. Yang, X.Y. et al. Superparamagnetic graphene oxide-Fe_3O_4 nanoparticles hybrid for controlled targeted drug carriers. *J. Mater. Chem.* **19**, 2710-2714 (2009).

31. Su, C.-Y. et al. High-quality thin graphene films from fast electrochemical exfoliation. *ACS Nano* **5**, 2332-2339 (2011).

32. Wang, J., Manga, K.K., Bao, Q. & Loh, K.P. High-yield synthesis of few-layer graphene flakes through electrochemical expansion of graphite in propylene carbonate electrolyte. *J. Am. Chem. Soc.* **133**, 8888-8891 (2011).

33. Parvez, K. et al. Electrochemically exfoliated graphene as solution-processable, highly conductive electrodes for organic electronics. *ACS Nano* **7**, 3598-3606 (2013).

34. Parvez, K. et al. Exfoliation of graphite into graphene in aqueous solutions of inorganic salts. *J. Am. Chem. Soc.* **136**, 6083-6091 (2014).

35. Xuhua, H. et al. Low defect concentration few-layer graphene using a two-step electrochemical exfoliation. *Nanotechnology* **26**, 105602 (2015).

36. Yang, S. et al. Organic radical-assisted electrochemical exfoliation for the scalable production of high-quality graphene. *J. Am. Chem. Soc.* **137**, 13927-13932 (2015).

37. Liu, J. et al. A green approach to the synthesis of high-quality graphene oxide flakes via electrochemical exfoliation of pencil core. *RSC Adv.* **3,** 11745-11750 (2013).

38. Wu, L. et al. Powder, paper and foam of few-layer graphene prepared in high yield by electrochemical intercalation exfoliation of expanded graphite. *Small* **10**, 1421-1429 (2014).

39. Parvez, K., Rincon, R.A., Weber, N.-E., Cha, K.C. & Venkataraman, S.S. One-step electrochemical synthesis of nitrogen and sulfur co-doped, high-quality graphene oxide. *Chem. Comm.* **52**, 5714-5717 (2016).

40. Yu, P. et al. Mechanically-assisted electrochemical production of graphene oxide. *Chem. Mater.* **28**, 8429-8438 (2016).

41. Gurzęda, B. et al. Synthesis of graphite oxide by electrochemical oxidation in aqueous perchloric acid. *Carbon* **100**, 540-545 (2016).

42. Tian, Z. et al. Facile electrochemical approach for the production of graphite oxide with tunable chemistry. *Carbon* **112**, 185-191 (2017).

43. Dimiev, A.M., Bachilo, S., Saito, R. & Tour, J.M. Reversible formation of ammonium persulfate sulfuric acid graphite intercalation compounds and their peculiar raman spectra. *ACS Nano* **6**, 7842-7849 (2012).

44. Dimiev, A.M. et al. Direct,real time monitoring of stage transitions in graphite intercalation compounds. *ACS Nano* **7**, 2773-2780 (2013).

45. Zhao, W., Tan, H.T., Liu, J. & Ferrari, A.C. Intercalation of few layer graphite flakes with $FeCl_3$: Raman determination of fermi level, layer by layer decoupling, and stability. *J. Am. Chem. Soc.* **133**, 5941-5946 (2011).

46. Dimiev, A.M. & Tour, J.M. Mechanism of graphene oxide formation. *ACS Nano* **8**, 3060-3068 (2014).

47. Kang, F., Zhang, T.Y. & Leng, Y. Electrochemical behavior of graphite in electrolyte of sulfuric and acetic acid. *Carbon* **35**, 1167-1173 (1997).

48. Rueffer, M., Bejan, D. & Bunce, N.J. Graphite: An active or an inactive anode? *Electrochim. Acta* **56**, 2246-2253 (2011).
