# Supplementary material for: Green synthesis of graphene oxide by seconds timescale water electrolytic oxidation
Source: Nat Commun. 2018 Jan 10;9:145. doi: 10.1038/s41467-017-02479-z (PMC5762692; doi:10.1038/s41467-017-02479-z)
Supplement: Supplementary file 3 — Description of Additional Supplementary Files [file 41467_2017_2479_MOESM3_ESM.pdf]

Description of Additional Supplementary Files

File Name: Supplementary Movie 1
Description: **GICP after EC intercalation**. GICP after EC intercalation in 98 wt. % sulfuric acid. The intercalated part keeps intact and shows a blue color after withdrawing from sulfuric acid.

File Name: Supplementary Movie 2
Description: **Continuous fast oxidation and exfoliation of GICP.** Continuous oxidation and exfoliation of a GICP slice in 50 wt. % sulfuric acid. The GICP slice is continuously introduced into the electrolyte with a specific dipping rate to keep a balance with the exfoliation rate. In this case, the GICP dipped in electrolyte continuously changes color from blue to yellow, expands to worm-like graphite oxide, and then exfoliates into the sulfuric acid solution. As a result, the GO sheets are produced continuously.

File Name: Supplementary Movie 3
Description: **Static oxidation of GICP in 50 wt. % H_2SO_4 solution.** Static oxidation and exfoliation of a GICP slice in 50 wt. % sulfuric acid. A GICP slice is dipped beneath liquid surface with an initial length of 1 cm without moving, and charged at +5 V. The dipped GICP is oxidized very quickly starting from the surface to the interior. Only after a few seconds, the GICP surface begins to become yellow from blue. With extending the reaction time, the yellow area gradually expands and the whole surface of the immersed GICP becomes yellow after about 60 s. Along with the oxidation and the production of a small amount of gas bubbles, the oxidized slice gradually exfoliates into the H_2SO_4 solution.

File Name: Supplementary Movie 4
Description: **Static reaction of FGP in 50 wt. % H_2SO_4 solution.** Static EC exfoliation of a FGP slice in 50 wt. % sulfuric acid. The part of FGP beneath liquid surface is about 1.5 cm in length, and charged at +5 V. The immersed FGP quickly exfoliates into the H_2SO_4 solution without visible color change along with the production of a large amount of gas bubbles.
