# Supplementary material for: Author Correction: Green synthesis of graphene oxide by seconds timescale water electrolytic oxidation
Source: Nat Commun. 2025 Oct 17;16:9212. doi: 10.1038/s41467-025-65021-6 (PMC12534571; doi:10.1038/s41467-025-65021-6)
Supplement: Supplementary file 1 — Supporting Information [file 41467_2025_65021_MOESM1_ESM.docx]

**Supporting Information**

**Background and motivation for XRD spectrum processing of deintercalated GICP**

- During our previous studies on graphite intercalation, we observed that the stage-I graphite intercalation compound paper (GICP) undergoes a color change from blue to light gray upon air exposure. Thus, in our studies related to the 2018 article, all GICP samples were stored in concentrated H₂SO₄ prior to characterization to prevent air-exposure-induced alterations.
- To reveal the structural changes caused by air exposure, we performed XRD measurements on two 2-hour-intercalated stage-I GICP samples at the same time in 2016: one was protected in concentrated H₂SO₄ (i.e., the intended GICP sample for the paper), while another was exposed in air for comparison. The sample exposed in air was not supposed to be included in our 2018 paper, and this comparative experiment was conducted purely due to scientific curiosity to investigate how air exposure affects the structure of GICP.
- As shown in Fig. S1, air exposure of the stage-I GICP sample (Fig. S1a) leads to distinct shifts in the XRD peaks compared to the 2-hour-intercalated sample (Fig. S1h). Specifically, the strongest peak and the second peak to its right shift toward higher angles, while the first peak to the right of the strongest peak shifts toward a lower angle. These observations suggest that air exposure induces deintercalation of GICP. For easy description, the strongest peak and the first and second peaks to its right for deintercalated sample are denoted as the (002), (003) and (004) peaks, respectively, same as the case of stage-I GICP.
- To deeply understand the structural changes induced by deintercalation, analysis of peak shape and width - in addition to peak position - is essential. Given the asymmetric shifts observed in the (002), (003), and (004) peaks, to better compare the changes in peak shape and width caused by deintercalation, one of the authors aligned the diffraction peaks of the deintercalated sample to those of the 2-hour-intercalated GICP sample by multiple peak shifting (Fig. S1).

**Detailed procedure for XRD spectrum processing of deintercalated GICP**

- **(002) peak alignment (Fig. S1b,c):** Copying the original XRD data of deintercalated GICP in the 19.86° - 31.48° range (Fig. S1b), and then using these data to replace the original XRD data in the 18.76°- 30.38° range (Fig. S1c), yielding a -1.1° (002) peak shift.
- **(003) peak alignment (Fig. S1d,e):** Copying the original XRD data of deintercalated GICP in the 29.36°- 34.36° range (Fig. S1d), and then using these data to replace the original XRD data in the 31.02° - 36.02° range (Fig. S1e), yielding a 1.66° (003) peak shift.

The above operations resulted in repetitive noises in the ranges of 28.26° - 30.38° and 31.02° - 33.14° (details see Fig. S2a).

- **(004) peak alignment (Fig. S1f,g):** Copying the original XRD data of deintercalated GICP in the 41.60° - 53.10° range (Fig. S1f), and then using these data to replace the original XRD data in the 38.90° - 50.40° range (Fig. S1g), yielding a -2.7° (004) peak shift. This operation resulted in repetitive noises in the ranges of 47.72° - 50.40° and 50.42° -53.10° (details see Fig. S2b).

After peak alignments, the processed XRD spectrum of deintercalated GICP (Fig. S1g) shows a profile nearly identical to that of 2-hour-intercalated GICP collected at the same time in 2016 (Fig. S1h), differing only slightly in peak shape and width. Because of this close visual similarity, we inadvertently included the processed deintercalated sample data instead of the correct 2-hour-intercalated GICP data during manuscript preparation.


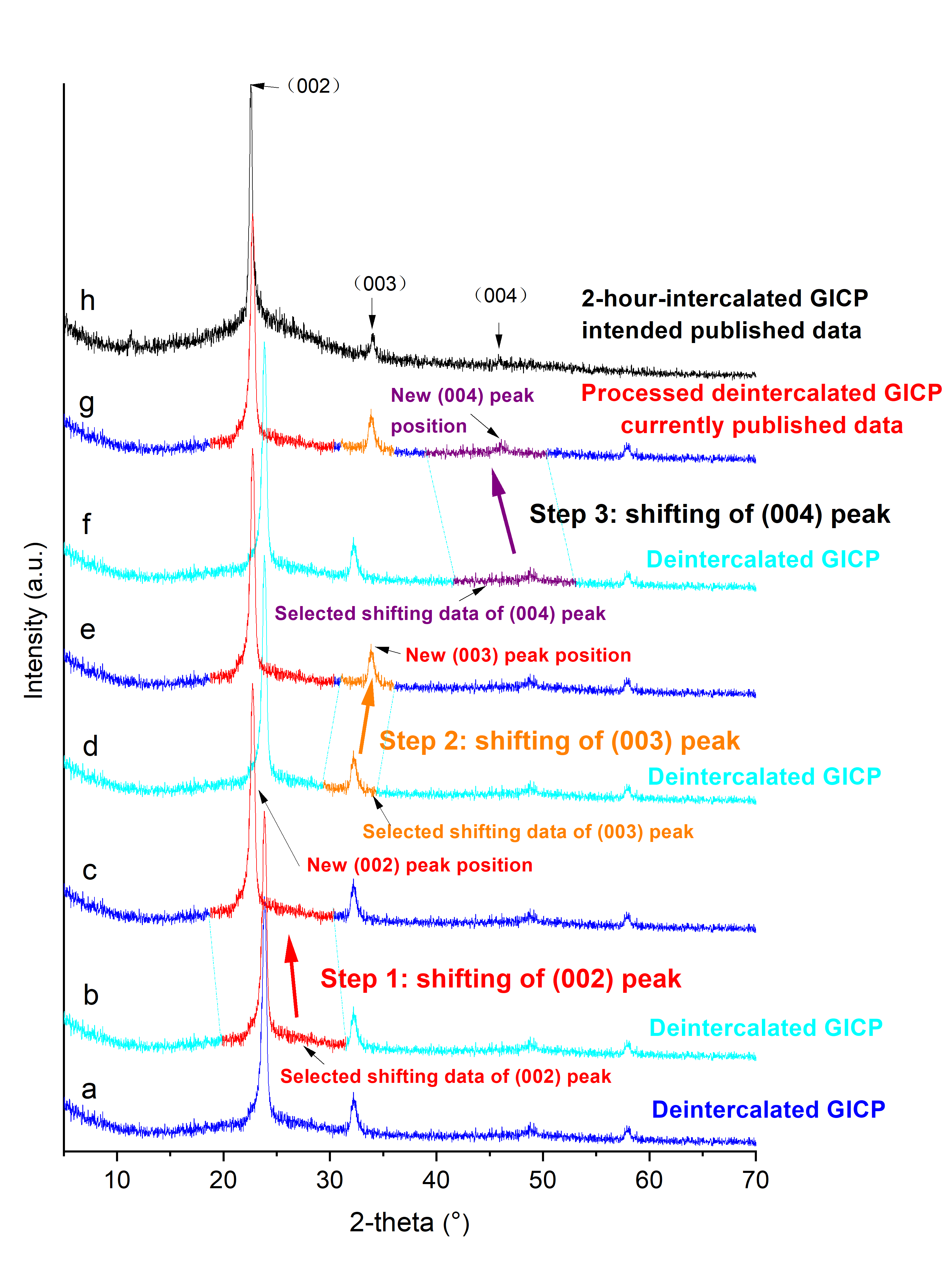


**Figure S1.** The detailed alignment procedure of the XRD spectrum of deintercalated GICP with that of 2-hour-intercalated GICP.


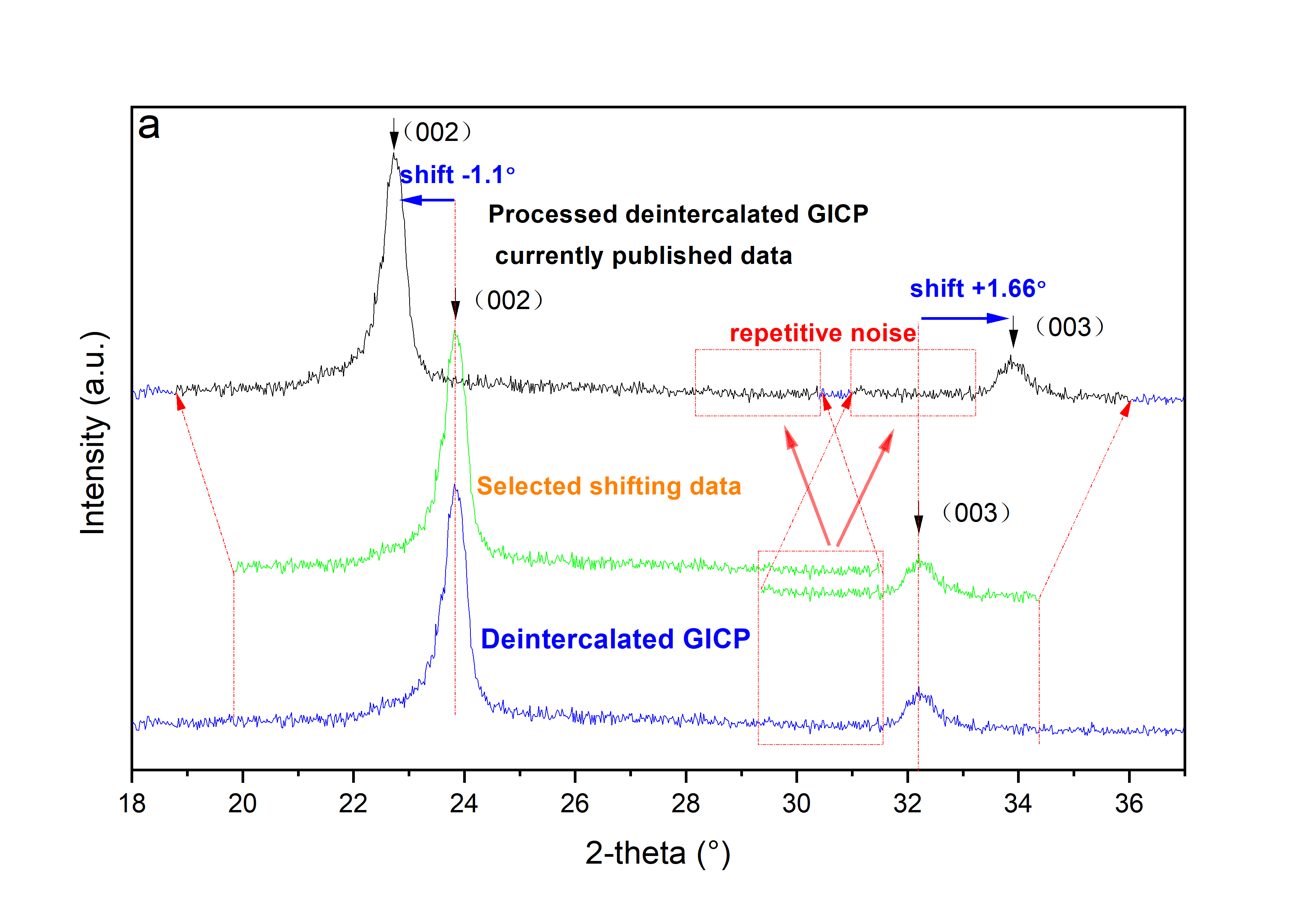

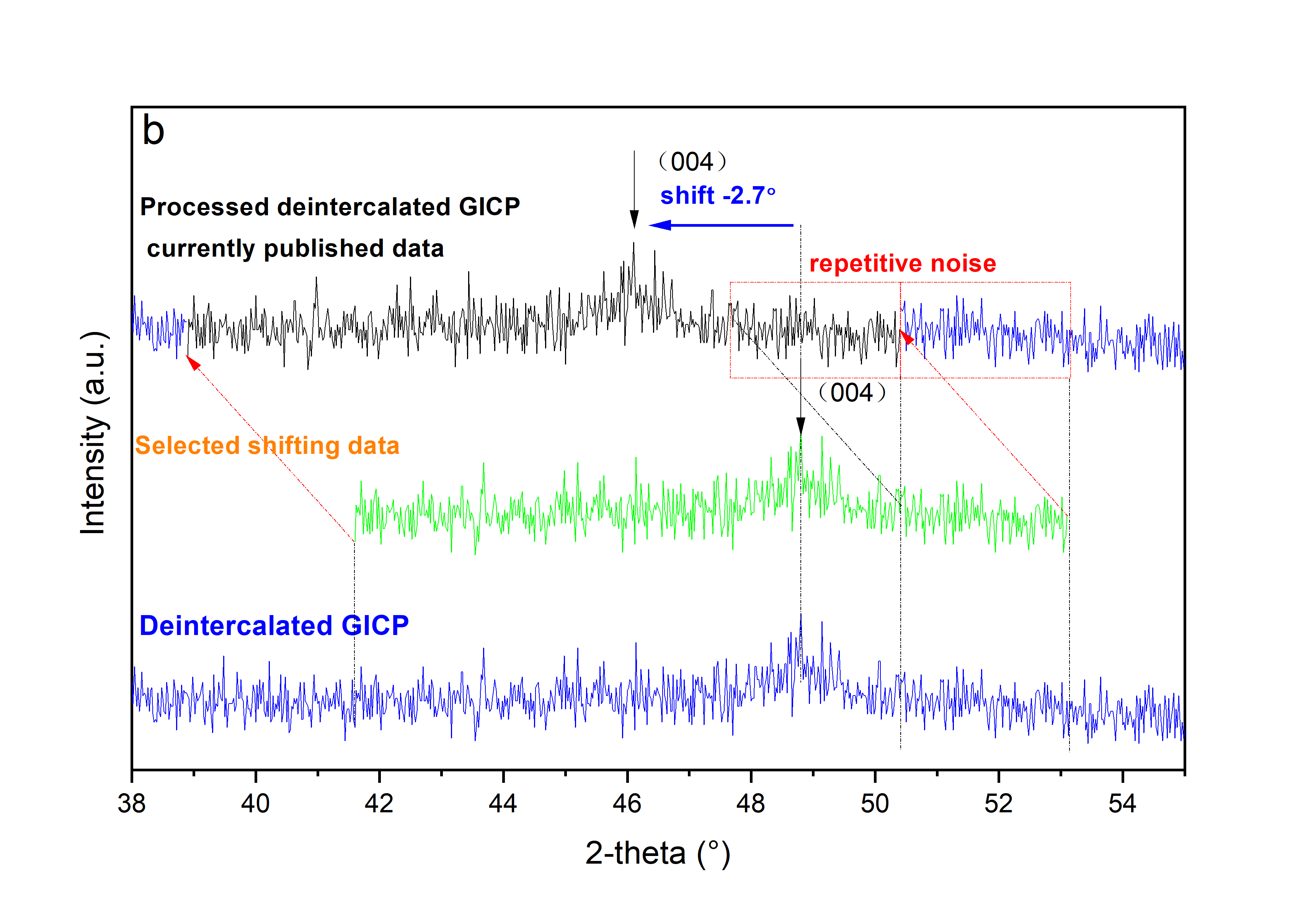


**Figure S2.** The generation of repetitive noises caused by the shifting of (002), (003), and (004) XRD peaks of deintercalated GICP during alignment processes.

**Results of the control experiments performed in 2025**

To further validate the reproducibility of our findings reported in the 2018 paper, we repeated XRD analyses of the 2-hour intercalation experiments on March 13 and 18, 2025. The newly obtained XRD spectra show excellent consistency with the original 2016 data (Fig. S3), reconfirming the conclusions drawn in the 2018 paper.


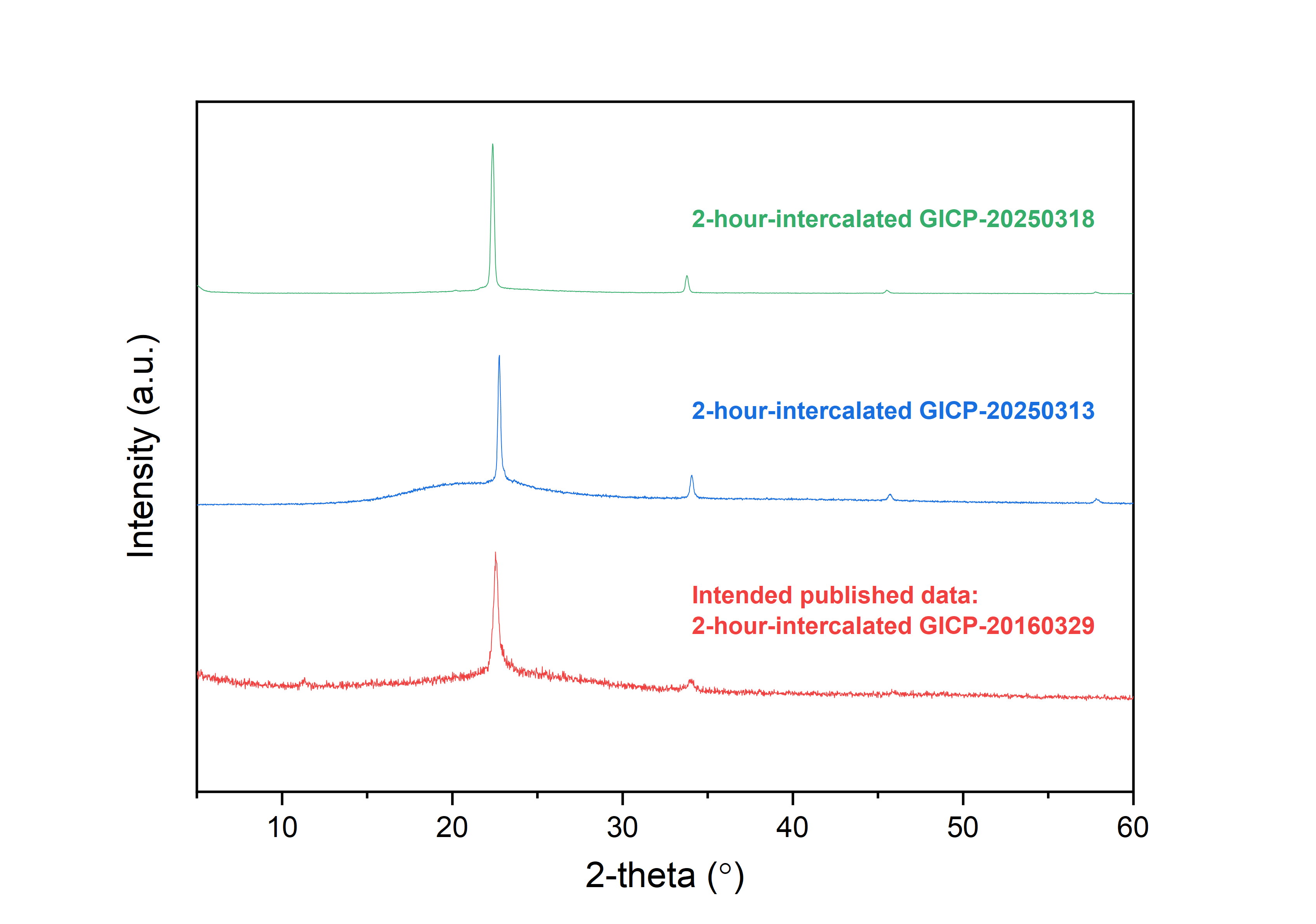


**Figure S3.** Comparison of XRD spectra of 2-hour-intercalated GICP samples that were synthesized and measured in 2016 and 2025.
